# Ciprofloxacin-Resistant *Salmonella enterica* Serovar Kentucky ST198 in Broiler Chicken Supply Chain and Patients, China, 2010–2016

**DOI:** 10.3390/microorganisms8010140

**Published:** 2020-01-19

**Authors:** Zhiying Xiong, Shaojun Wang, Yumei Huang, Yuan Gao, Haiyan Shen, Zhengquan Chen, Jie Bai, Zeqiang Zhan, Junping Wen, Ming Liao, Jianmin Zhang

**Affiliations:** 1 National and Regional Joint Engineering Laboratory for Medicament of Zoonoses Prevention and Control, Lingnan Guangdong Laboratory of Modern Agriculture, College of Veterinary Medicine, South China Agricultural University, Guangzhou 510642, China; xiongzhiying99@163.com (Z.X.); wShaojun_1@163.com (S.W.); hym90128@163.com (Y.H.); tianc__001@163.com (Y.G.); czquan941111@163.com (Z.C.); 13538827914@163.com (J.B.); zeqiangzhan_v@163.com (Z.Z.); JunpingWen@126.com (J.W.); 2 Institude of Animal Health, Guangdong Academy of Agricultural Sciences, Guangzhou 510640, China; haiyan_0001@163.com

**Keywords:** *Salmonella*, Kentucky, mutation, ciprofloxacin resistance, pulsed-field gel electrophoresis

## Abstract

*Salmonella enterica* serovar Kentucky (*S*. Kentucky) sequence type 198 has emerged as a global zoonotic pathogen. We explored *Salmonella enterica* serovar Kentucky ST198 samples from the broiler chicken supply chain and patients between 2010 and 2016. Here, we collected 180 *S*. Kentucky isolates from clinical cases and the poultry supply chain. We performed *XbaI* pulsed-field gel electrophoresis and multilocus sequence typing. We assessed mutations in the quinolone resistance-determining regions and screened for the presence of the *Salmonella* genomic island 1 (SGI1). We determined that 63 (35.0%) of the 180 isolates were *S*. Kentucky ST198. Chinese strains of *S*. Kentucky ST198 have a high transmission of ciprofloxacin resistance (38/63, 60.3%) and a high risk of multidrug resistance. The quinolone resistance of the *S*. Kentucky ST198 strain found in China may be due to mutations in its quinolone resistance-determining region. Our study firstly revealed that ciprofloxacin-resistant *S*. Kentucky ST198 strains can undergo cross-host transmission, thereby causing a serious foodborne public health problem in China.

## 1. Introduction

*Salmonella enterica* is a major global foodborne pathogen [1]. To date, more than 2600 *Salmonella* serovars have been identified worldwide [2]. There are an estimated 93.8 million cases of salmonellosis worldwide [3]. In the United States, salmonellosis is the second leading cause of foodborne illnesses in humans [4], accounting for approximately 1 million cases, 19,336 hospitalizations, and 378 deaths each year. Approximately 31% of all foodborne-related deaths that occur each year in the USA are attributed to *Salmonella* infections, and the annual medical cost for salmonellosis in the USA is estimated at $3.7 billion. In China, approximately 70% to 80% of foodborne disease outbreaks are caused by *Salmonella*, and most of these disease outbreaks are associated with the ingestion of contaminated livestock and poultry products [5]. Food of animal origin, particularly chicken, is considered to be a major reservoir of *Salmonella* [6].

*Salmonella enterica* serotype Kentucky (*S*. Kentucky) is one of the serovars most frequently isolated from poultry carcasses in the USA [7]. Historically, *Salmonella enterica* serotype Kentucky has rarely been associated with human illness. However, the ciprofloxacin-resistant *Salmonella enterica* subsp. *enterica* serovar Kentucky sequence type 198 (ST198) has emerged as a global human pathogen [8]. Human illnesses caused by this pathogen in North America and Europe are typically associated with a history of travel to Africa, Southeast Asia, and the Middle East, where this pathogen is established in poultry [9,10]. Ciprofloxacin-resistant *S*. Kentucky ST198 is also established in poultry in France [9], Poland, and other European countries, and this organism represents a significant risk to public health and food safety [8,9]. However, in China, there is currently no systematic study of the epidemiology and drug resistance of *S*. Kentucky. Here, we have systematically studied the epidemiology and molecular characteristics of drug resistance in *S*. Kentucky ST198 isolates in China, which can support basic research, clinical practice, and public health risk forecasts for *Salmonella*.

## 2. Materials and Methods

### 2.1. Sample Collection Isolate Identification

Between 2010 and 2016, *Salmonella* strains were collected from 23 provinces, including Shanghai, Guangxi, Hubei, Chongqing, Fujian, Xinjiang, and Guangdong. Clinical *Salmonella* strains were provided by the provincial Centers for Disease Control and regional hospitals. Food and environmental samples were collected from a retail market. *Salmonella* isolation and identification was performed according to the US FDA Bacteriological Analytical Manual, as previously described, with some modifications [8,9,11,12]. The O and H antigens were characterized using slide agglutination with hyperimmune sera (S&A Reagents Lab, Bangkok, Thailand), and the serotype was assigned following the manufacturer’s instructions [13].

### 2.2. Polymerase Chain Reaction (PCR) Amplification and Multilocus Sequence Typing (MLST)

Genomic DNA was isolated using the InstaGene Matrix (Bio-Rad, Hercules, CA, USA) according to the manufacturer’s protocol. All primer sequences for amplification and sequencing were obtained from the MLST Databases at the University of Warwick (www.mlst.warwick.ac.uk/mlst/dbs/Senterica). The PCR cycling conditions were as indicated in instructions posted on the website. PCR products were purified with Sephadex G-50 fine resin (GE Healthcare Bio-Sciences AB, Uppsala, Sweden). Nucleotide cycle-sequencing was performed directly on purified PCR templates using automated Sanger dideoxychain termination methods and the primers described on the MLST website. Sequences of seven housekeeping genes (*aroC*, *dnaN*, *hemD*, *hisD*, *purE*, *sucA*, and *thrA*) were compared with the available MLST database (http://mlst.warwick.ac.uk/mlst/dbs/Senterica) to get the allele number and sequence typing (ST) number for each isolate. Sequence information for newly assigned alleles and STs was deposited in the MLST database [14].

### 2.3. Antimicrobial Susceptibility Testing

The antimicrobial susceptibility of all strains was determined using standard agar dilution method on Mueller–Hinton agar (MH) [15]. The following antibiotics (Oxoid, UK) were used: ampicillin, nalidixic acid, ciprofloxacin, ofloxacin, ceftazidime, cefquinome, gentamicin, amikacin, chloramphenicol, florfenicol, polymyxin B, imipenem, sulfafurazole, and tetracycline. *Escherichia coli* ATCC strain 25922 served as the control. Results were interpreted based on the Clinical and Laboratory Standards Institute guidelines (CLSI, 2013).

### 2.4. Detection of Quinolone Resistance Genes, QRDR Mutations, and SGI1 via PCR

Four fluoroquinolone regulatory genes (*gyrA*, *gyrB*, *parC*, and *parE*) were analyzed using PCR and sequencing. Plasmid-mediated resistance genes, including *oqxAB*, *qepA*, *aac(6′)-Ib*, *qnrS*, *qnrD*, *qnrC*, *qnrB*, and *qnrA* (fluoroquinolone resistance), were analyzed via PCR [15]. According to the sequence of SGI1-Ks, SGI1-Ps, SGI1-Ps-Qs, SGI1-Qs synthetic primers (SGI1-Ks: forward: 5′-AAGGATTTCCTGACCCTG-3′, reverse: 5′-ATGGATGTGGTGGCTGAAGG-3′; SGI1-Ps: forward: 5′-CGGTTTTGAATAAGAAGGCA-3′, reverse: 5′-CCAATGCTTAATCAGTGAGG-3′; SGI1-Ps-Qs: forward: 5′-ATGAGTATTCAACATTTCCG-3′, reverse: 5′-GTATTGTCGTCGGGATGATT-3′; SGI1-Qs: forward: 5′-CGGTTTTGAATAAGAAGGCA-3′, reverse: 5′-GTATTGTCGTCGGGATGATT-3′.), the total DNA of *Salmonella* was used as a template for PCR amplification, and the amplified products were detected by agarose gel electrophoresis [16].

### 2.5. Pulsed-Field Gel Electrophoresis

*S*. Kentucky isolates were subtyped via pulsed-field gel electrophoresis (PFGE) [17] to determine their genetic relatedness according to the Pulse-Net protocol. PFGE was performed after digestion of genomic DNA with the restriction enzyme *XbaI*. *Salmonella* H9812 was used as the standard control strain. PFGE results were analyzed by BioNumerics software (Version 5.1; Applied-Maths, Sint-Martens-Latem, Belgium).

## 3. Results

### 3.1. Prevalence of Salmonella enterica Kentucky

A total of 180 strains of *S.* Kentucky were identified: 105 (0.9%) of 12,011 clinical *Salmonella* strains isolated from 15 provinces (including Shanghai, Guanxi, Hubei, Chongqing, Fujian, and Xinjiang), and 75 (1.8%) of 4236 chicken and environmental samples from eight provinces (including Guangdong, Hong Kong, Fujian, and ShenZheng). Of the 180 isolates of *S.* Kentucky obtained in China, 63 (35.0%) were *S.* Kentucky ST198, consisting of 40 strains from patient fecal samples, 18 strains from chicken, and 5 strains from environmental sources (e.g., vegetables or river water).

### 3.2. Antimicrobial Susceptibility Testing

**Figure 1:**
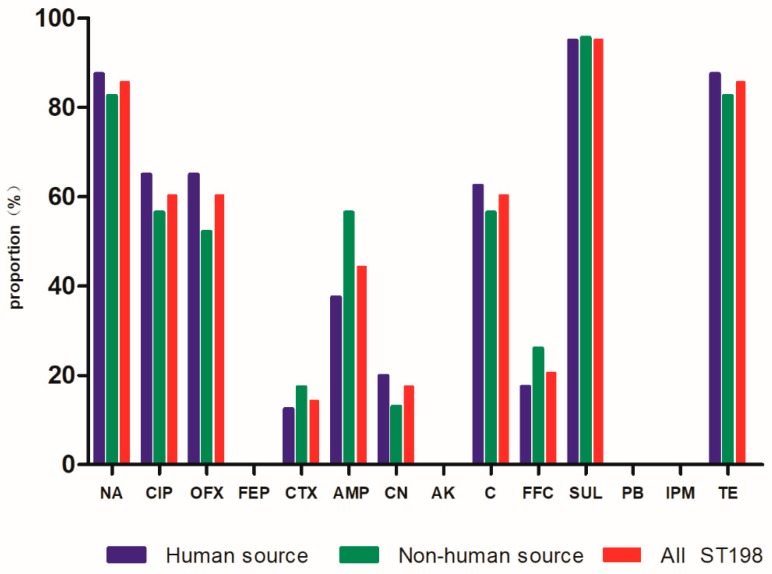
Drug resistance rate of 63 strains of *Salmonella* Kentucky ST198. Abbreviations: Penicillins: AMP (ampicillin); Quinolones: NA (nalidixic acid), CIP (ciprofloxacin), OFX (ofloxacin); Cephalosporins: FEP (cefquinome), CTX (cefotaxime); Aminoglycosides: CN (gentamicin), AK (amikacin); Chloram phenicols: C (chloramphenicol), FFC (florfenicol); Polymyxins: PB (polymyxin B); Carbapenems: IPM (imipenem); Sulfonamides: SUL (sulfafurazole); Tetracyclines: TE (tetracycline).

**Figure 2:**
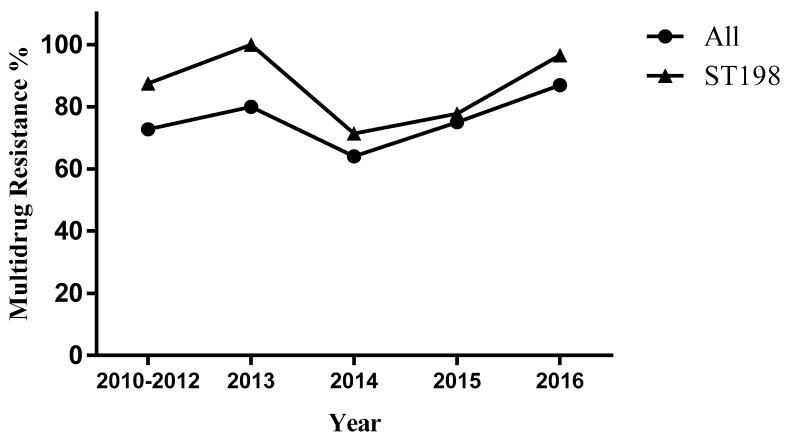
Multidrug resistance of *Salmonella enterica* serovars Kentucky, China, 2010–2016.

Among the 63 *S*. Kentucky ST198 isolates, the analysis of antimicrobial susceptibility testing (Figure 1) showed a high proportion of *S*. Kentucky ST198 isolates were resistant to fluoroquinolones (ciprofloxacin 60.3%, ofloxacin 60.3%, and nalidixic acid 85.7%), 28 (44.4%) of the 63 isolates were resistant to ampicillin, and 9 (14.3%) of the 63 isolates were resistant to cephalosporin. We defined strains resistant to three or more antibiotics as multidrug-resistant strains. For *S.* Kentucky ST198 strains and for all strains of *S*. Kentucky, the rate of multidrug resistance increased from 2014 to 2015 and again in 2016 (Figure 2, Table S1).

### 3.3. Detection of Quinolone Resistance Genes

*S*. Kentucky ST198 strains were found to have mutations in the *gyrA* (His78Asn, Ser83Phe, Asp87Asn, Asp87Gly, Asp87Tyr) and *parC* (Tyr57Ser, Ser80Ile) genes (Table 1). Statistical analysis was performed using R Studio version 1.0.143, and statistical regression was performed using a linear regression test. We considered a difference significant if the *p* value was <0.05. We found the *gyrA* Ser83Phe and Asp87Gly mutations played a significant role in acquisition by *S.* Kentucky ST198 of nalidixic resistance (*p* < 0.05), and the *parC*(Ser80Ile) mutation could give rise to resistance to ciprofloxacin (*p* < 0.05) and ofloxacin (*p* < 0.05). Here, *S*. Kentucky ST198 strains carried four plasmid-mediated quinolone resistance genes: *aac(6′)-Ib-cr* (30.2%), *oqxAB* (4.8%), *qnrS* (7.9%), and *qnrB* (6.3%).

We tested the SGI1 type of the 63 *S*. Kentucky strains, mainly to study SGI1-Ks and its derivatives of the *S*. Kentucky ST198 strain in China. We found that SGI1-Ks and its derivatives were detected in 28.6% of *S*. Kentucky ST198 strains in China (Table 2, Table S2).

### 3.4. Pulsed-Field Gel Electrophoresis (PFGE)

**Figure 3:**
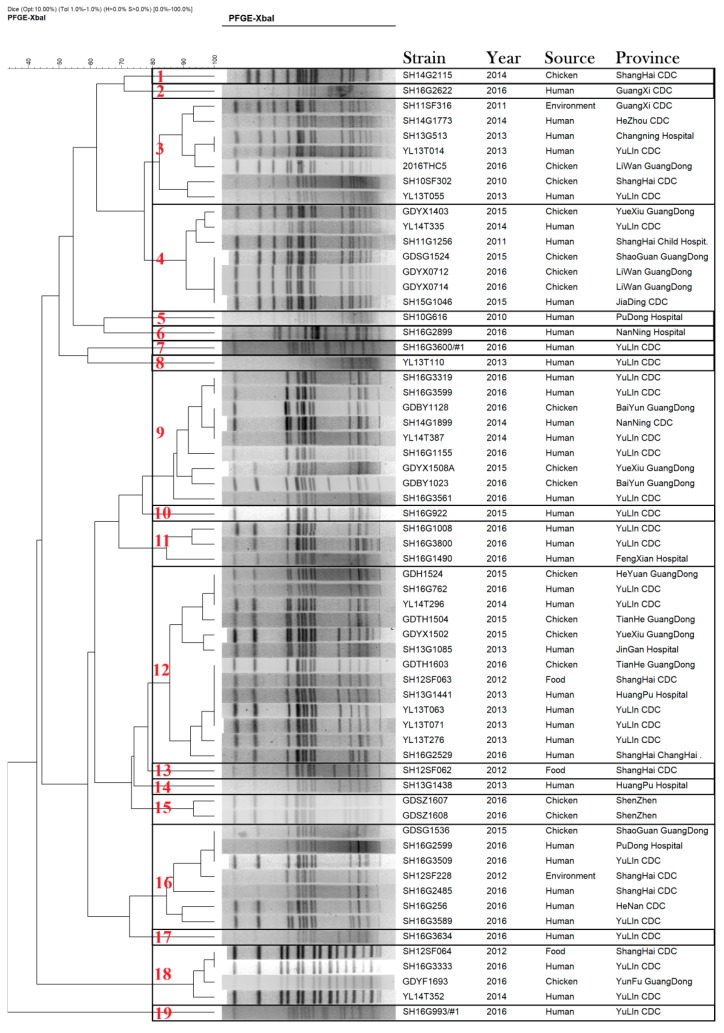
Dendrogram of 63 pulsed-field gel-electrophoresis-based profiles of *Salmonella enterica* serovar Kentucky ST198 strains isolated from human, environmental, and chicken samples in China, 2010–2016.

All *S.* Kentucky ST198 isolates were typed using PFGE with XbaI restriction enzyme. The 63 *S.* Kentucky ST198 strains isolated from different sources were clustered into 19 unique profiles with a percentage similarity >80%. According to Figure 3, there were 11 profiles with only one isolate and 8 profiles with 2–13 isolates each. Of 40 case-patients in China, we found 10 case-patients were linked to *S.* Kentucky ST198 strains from chicken, in that they had the same clone. Samples from 24 case-patients were collected from YuLin Centers for Disease Control, and the isolates were distributed diffusely across 12 unique PFGE profiles.

## 4. Discussion

*Salmonella* is a pathogenic microorganism that poses a serious threat to public health safety [17,18]. *S.* Kentucky has been closely related to poultry since it was first identified in 1937, and it has been communicated between humans and livestock throughout the world [19]. *S*. Kentucky has attracted much attention in Europe, America, Africa, and elsewhere [20]. However, there have not been any previous reports on specific sequence types of *S.* Kentucky in China before 2018. In our study, it was found that *S*. Kentucky had a certain prevalence in different parts of China, while ST198 (63/180, 35.0%) was the main ST type from different hosts in Shanghai, Guangdong, Guangxi, Henan, and Xinjiang (including human feces samples, chicken, and environmental samples). This means that ST198 has caused a public health threat in China that needs to be taken seriously. Thus, for food safety and preventive public health measures, it is important that we have presented the first report on the prevalence and resistance characteristics of ST198 in China.

Recently, *S*. Kentucky has spread widely around the world, which has conferred multidrug resistance under the stress of antibiotics [21,22]. In this study, we further analyzed the antimicrobial susceptibility and PFGE typing of *S.* Kentucky ST198 that were isolated from different provinces and regions in China from 2010 to 2016. Global spread of mobile antimicrobial drug resistance determinants in human and animal *Escherichia coli* and *Salmonella* strains causing community-acquired infections [23,24,25]. It has been shown that *S*. Kentucky has been increasing its multidrug resistance rate since 2014. Fluoroquinolones are the preferred method of clinical treatment for nontyphoid salmonellosis [26]; at the same time, they are the main reasons for the spread of ST198 in the world [27]. The most important targets of fluoroquinolones for microorganisms are DNA gyrase (*gyrA* and *gyrB*) and topoisomerase IV (*parC* and *parE*); the mutations in the *gyrA* and *parC* genes are the most common mechanisms of bacterial resistance to quinolone antibiotics generally [28]. It is noteworthy that that the proportion of *S*. Kentucky ST198 isolates that were resistant to fluoroquinolone was high, with resistance rates greater than 60.0%. Statistical analysis revealed that the mutations of *gyrA* (Ser83Phe, Asp87Gly) and *parC* (Ser80Ile) have a major influence on the high level of drug resistance of quinolones (ciprofloxacin, nalidixic acid, and ofloxacin). As can be seen in this study, quinolone resistance caused by the quinolone resistance-determination region mutation was the same as the internationally common ST198 resistance mechanism [26,27]. It is well known that quinolone-resistant ST198 is widespread in China, both in human and livestock sources.

Our study displayed that the ciprofloxacin-resistant *S.* Kentucky ST198 strain can spread among broiler supply chain and patients, which has been involved in cross-host transmission in China. The study found a higher proportion of multidrug resistance among *S.* Kentucky ST198 isolates than the average over all *S.* Kentucky strains in China (Figure 2). The multidrug resistance region in SGI1 can incorporate new DNA segments in the same way as multiple antibiotic resistance regions in plasmids, thereby providing conditions for the spread of drug resistance and acquired drug resistance [29]. Notably, the presence of quinolone plasmid resistance genes and virulence islands has made the spread of drug resistance and acquired resistance more complicated, and different PFGE profiles contain diverse molecular characteristics of *S.* Kentucky ST198 strains in China.

## 5. Conclusions

In conclusion, this is the first report about the prevalence of ciprofloxacin-resistant *S.* Kentucky ST198 through the broiler supply chain and patients in China. High risks for development of multidrug resistance make infections with ciprofloxacin-resistant *S.* Kentucky ST198 a serious public health concern in China.

## Figures and Tables

**Table 1:** Drug resistance genes detected in strains of *Salmonella* Kentucky ST198.

Fluoroquinolone Resistance Classification		Gene about Fluoroquinolones	Proportion (%)
PMQR (plasmid-mediated quinolone resistance)		*oqxAB*	4.8%(3/63)
		*qepA*	0.0%(0/63)
		*aac(6′)-Ib-cr*	30.2%(19/63)
		*qnrS*	7.9%(5/63)
		*qnrD*	0.0%(0/63)
		*qnrC*	0.0%(0/63)
		*qnrB*	6.3%(4/63)
		*qnrA*	0.0%(0/63)
QRDR (quinolone resistant determining region)	*gyrA*	His78Asn	3.2%(2/63)
		Ser83Phe	87.3%(55/63)
		Asp87Asn	47.6%(30/63)
		Asp87Gly	12.7%(8/63)
		Asp87Tyr	1.6%(1/63)
	*parC*	Ser85Ile	65.1%(41/63)

**Table 2:** Rate of detection of *Salmonella* multidrug-resistant genomic island 1 in *Salmonella* Kentucky ST198.

*Salmonella* Multidrug-Resistant Genomic Island 1 (SGI1)	Detection Rate (%)
SGI1-Ks	28.6% (18/63)
SGI1-Ps	1.6% (1/63)
SGI1-Ps-Qs	0.0% (0/63)
SGI1-Qs	0.0% (0/63)

## Supplementary Materials

Supplementary materials can be found at https://www.mdpi.com/2076-2607/8/1/140/s1.
