# Supplementary material for: Ciprofloxacin-Resistant Salmonella enterica Serovar Kentucky ST198 in Broiler Chicken Supply Chain and Patients, China, 2010–2016
Source: Microorganisms. 2020 Jan 19;8(1):140. doi: 10.3390/microorganisms8010140 (PMC7022574; doi:10.3390/microorganisms8010140)
Supplement: Supplementary file 1 [file microorganisms-08-00140-s001.pdf]

Table S1. The minimum inhibitory concentration (MIC) and multi-drug resistance of *Salmonella* Kentucky ST198.

Strain	Year	Source	Province	MIC(μg/mL) resistant breakpoint of fourteen antibiotics														MDR
				NA (≥32μg/mL)	CIP (≥1μg/mL)	OFX (≥8μg/mL)	FEP (≥16μg/mL)	CTX (≥4μg/mL)	AMP (≥32μg/mL)	CN (≥16μg/mL)	AK (≥64μg/mL)	C (≥32μg/mL)	FFC (≥16μg/mL)	SUL (≥512μg/mL)	PB (≥8μg/mL)	IPM (≥4μg/mL)	TE (≥16μg/mL)	
SH14G2115	2014	Chicken	ShangHai CDC	512	16	8	0	0.125	0	8	2	512	8	512	0.5	1	128	NA+CIP+OFX+C+SUL+TE
SH16G2622	2016	Human	Guangxi CDC	4	0.125	0.25	0.125	1	4	1	4	8	4	512	0.5	1	4	SUL
SH11SF316	2011	Environment	Guangxi CDC	4	0	1	0	0.125	2	4	0	512	4	512	0.25	0.25	16	C+SUL+TE
SH14G1773	2014	Human	HeZhou CDC	4	0.125	0.125	0.125	1	8	1	2	8	8	256	0.25	1	4	
SH13G513	2013	Human	Changning Hospital	512	0.25	1	0	0.125	2	8	2	256	4	512	0.5	0.25	16	NA+C+SUL+TE
YL13T014	2013	Human	YuLin CDC	512	0.25	1	0	0.125	0	8	1	256	4	512	0.5	0.25	16	NA+C+SUL+TE
2016THC5	2016	Chicken	LiWan GuangDong	512	0.5	1	0	0.125	512	1	2	4	4	512	0.5	0.5	128	NA+AMP+SUL+TE
SH10SF302	2010	Chicken	ShangHai CDC	512	16	0.5	0	0.125	8	8	1	512	8	512	0.5	0.25	64	NA+CIP+C+SUL+TE
YL13T055	2013	Human	YuLin CDC	512	0.25	1	0	0.125	0	8	2	256	8	512	0.5	0.25	16	NA+C+SUL+TE
GDYX1403	2015	Chicken	YueXiu GuangDong	512	0.5	2	0	0.25	2	1	2	4	2	512	0.5	0.5	8	NA+SUL
YL14T335	2014	Human	YuLin CDC	512	0.25	1	0	0.125	0	8	1	512	4	512	0.5	0.25	16	NA+C+SUL+TE
SH11G1256	2011	Human	ShangHai Child Hospit.	512	0.25	1	0	0.125	2	8	2	512	4	512	0.5	0.25	16	NA+C+SUL+TE
GDSG1524	2015	Chicken	ShaoGuan GuangDong	512	0.5	2	0	0.25	512	1	2	4	2	512	0.5	0.5	64	NA+AMP+SUL+TE
GDYX0712	2016	Chicken	LiWan GuangDong	512	0.5	1	0	0.125	512	1	2	4	4	512	0.5	0.5	128	NA+AMP+SUL+TE
GDYX0714	2016	Chicken	LiWan GuangDong	512	0.5	1	0	0.125	512	0.5	2	4	4	512	0.5	0.5	128	NA+AMP+SUL+TE
SH15G1046	2015	Human	JiaDing CDC	512	16	8	0	0.125	64	8	1	512	4	512	0.5	0.25	128	NA+CIP+OFX+AMP+C+SUL+TE
SH10G616	2010	Human	PuDong Hospital	512	0.5	1	0	0.125	2	8	2	512	4	512	0.5	0.25	16	NA+C+SUL+TE
SH16G2899	2016	Human	NanNing Hospital	512	0.5	2	0	0.25	512	32	4	256	256	512	0.5	0.5	64	NA+AMP+CN+C+FFC+SUL+TE
SH16G3600/#1	2016	Human	YuLin CDC	16	0.5	1	0.125	1	4	1	2	512	256	512	0.5	1	128	C+FFC+SUL+TE
YL13T110	2013	Human	YuLin CDC	512	0.25	1	0	0.125	0	8	1	256	4	512	0.5	0.25	16	NA+C+SUL+TE
SH16G3319	2016	Human	YuLin CDC	512	32	16	0	0.25	2	1	4	128	256	512	0.5	0.5	64	NA+CIP+OFX+C+FFC+SUL+TE
SH16G3599	2016	Human	YuLin CDC	512	64	16	0	0.25	2	1	2	4	4	512	0.5	0.5	64	NA+CIP+OFX+SUL+TE
GDBY1128	2016	Chicken	BaiYun GuangDong	16	0.5	2	0	0.25	2	1	2	256	256	512	0.5	0.5	128	C+FFC+SUL+TE
SH14G1899	2014	Human	NanNing CDC	4	0	0	0	0.125	0	0	0	512	4	8	0.25	0.25	16	C+TE
YL14T387	2014	Human	YuLin CDC	512	0.5	8	0	0.125	0	8	2	512	4	512	0.5	0.25	128	NA+OFX+C+SUL+TE
SH16G1155	2016	Human	YuLin CDC	512	32	16	0	0.125	2	1	4	4	8	512	0.5	0.5	128	NA+CIP+OFX+SUL+TE
GDYX1508A	2015	Chicken	YueXiu GuangDong	16	0.5	2	0	0.25	2	1	2	256	256	512	0.5	0.5	64	C+FFC+SUL+TE
GDBY1023	2016	Chicken	BaiYun GuangDong	512	128	64	0	0.5	512	64	2	256	256	512	0.5	0.5	128	NA+CIP+OFX+AMP+CN+C+FFC+SUL+TE
SH16G3561	2016	Human	YuLin CDC	512	32	16	0	0.125	2	1	4	4	4	512	0.5	0.5	128	NA+CIP+OFX+SUL+TE
SH16G922	2015	Human	YuLin CDC	512	64	16	8	32	512	1	2	2	2	512	0.5	0.5	0	NA+CIP+OFX+CTX+AMP+SUL
SHG1008	2016	Human	YuLin CDC	512	32	16	0	0.125	2	1	4	4	8	512	0.5	0.5	128	NA+CIP+OFX+SUL+TE
SH16G3800	2016	Human	YuLin CDC	512	64	16	0	2	1	64	2	256	128	512	0.5	1	32	NA+CIP+OFX+CTX+CN+C+FFC+SUL+TE
SH16G1490	2016	Human	FengXian Hospital	512	32	16	0.125	1	512	1	2	8	4	512	0.5	1	4	NA+CIP+OFX+AMP+SUL
GDTH1524	2015	Chicken	HeYuan GuangDong	512	64	16	0	0.25	2	1	2	2	2	512	0.5	0.5	64	NA+CIP+OFX+SUL+TE
SH16G762	2016	Human	YuLin CDC	16	0.5	2	0	0.25	2	1	2	256	256	512	0.5	0	64	C+FFC+SUL+TE
YL14T296	2014	Human	YuLin CDC	512	16	8	0	0.125	0	8	1	512	4	512	0.25	0.25	128	NA+CIP+OFX+C+SUL+TE
GDTH1504	2015	Chicken	TianHe GuangDong	512	64	16	8	0.25	2	1	2	4	2	512	0.5	0.5	64	NA+CIP+OFX+SUL+TE
GDXY1502	2015	Chicken	YueXiu GuangDong	512	0.5	1	0	0.25	2	1	2	4	2	512	0.5	0.5	0	NA+SUL
SH13G1085	2013	Human	JinGan Hospital	512	16	8	0.125	0.125	64	8	2	512	4	512	0.5	0.25	128	NA+CIP+OFX+AMP+C+SUL+TE
GDTH1603	2016	Chicken	TianHe GuangDong	512	32	16	2	2	512	1	2	4	4	512	0.5	0.5	128	NA+CIP+OFX+CTX+AMP+SUL+TE
SH12SF063	2012	Food	ShangHai CDC	512	16	8	0.125	0.125	64	8	2	512	4	512	0.5	0.25	128	NA+CIP+OFX+AMP+C+SUL+TE
SH13G1441	2013	Human	HuangPu Hospital	512	16	8	0.125	0.125	128	8	2	512	4	512	0.5	0.25	128	NA+CIP+OFX+AMP+C+SUL+TE
YL13T063	2013	Human	YuLin CDC	512	16	8	0.125	0.125	64	8	2	512	4	512	0.5	0.25	128	NA+CIP+OFX+AMP+C+SUL+TE
YL13T071	2013	Human	YuLin CDC	512	16	8	0.125	0.125	64	16	2	512	4	512	0.5	0.25	128	NA+CIP+OFX+AMP+CN+C+SUL+TE
YL13T276	2013	Human	YuLin CDC	512	16	8	0	0.125	0	16	2	512	8	512	0.5	0.25	128	NA+CIP+OFX+CN+C+SUL+TE
SH16G2529	2016	Human	ShangHai ChangHai	512	16	8	0	0.25	2	1	2	4	4	512	0.5	0.5	8	NA+CIP+OFX+SUL
SH12SF062	2012	Food	ShangHai CDC	512	16	8	0.125	0.125	64	8	2	512	4	512	0.5	0.25	128	NA+CIP+OFX+AMP+C+SUL+TE
SH13G1438	2013	Human	HuangPu Hospital	512	16	8	0.125	0.125	64	8	2	512	4	512	0.5	0.25	128	NA+CIP+OFX+AMP+C+SUL+TE
GDSZ1607	2016	Chicken	ShenZhen	512	128	16	4	32	512	8	4	128	256	512	0.5	0.5	8	NA+CIP+OFX+CTX+AMP+C+FFC+SUL
GDSZ1608	2016	Chicken	ShenZhen	512	128	16	4	32	512	8	2	128	256	512	0.5	0.5	8	NA+CIP+OFX+CTX+AMP+C+FFC+SUL
GDSG1536	2015	Chicken	ShaoGuan GuangDong	512	64	16	8	32	512	32	2	4	4	512	0.5	0..5	64	NA+CIP+OFX+CTX+AMP+CN+SUL+TE
SH16G2599	2016	Human	PuDong Hospital	512	64	16	8	32	512	32	2	2	2	512	1	0.5	64	NA+CIP+OFX+CTX+AMP+CN+SUL+TE
SH16G3509	2016	Human	YuLin CDC	512	32	16	0	0.125	2	1	4	4	8	512	0.5	0.5	128	NA+CIP+OFX+SUL+TE
SH12SF228	2012	Environment	ShangHai CDC	4	0	0	0	0.5	0	0	0	512	4	8	0.25	0.25	16	C+TE
SH16G2485	2016	Human	ShangHai CDC	512	32	16	8	32	512	32	2	2	4	512	0.5	0.5	64	NA+CIP+OFX+CTX+AMP+CN+SUL+TE
SH16G256	2016	Human	HeNan CDC	512	64	16	8	32	512	64	4	4	4	512	0.5	0.5	64	NA+CIP+OFX+CTX+AMP+CN+SUL+TE
SH16G3589	2016	Human	YuLin CDC	512	64	16	8	32	512	32	2	4	4	512	0.5	0.5	64	NA+CIP+OFX+CTX+AMP+CN+SUL+TE
SH16G3634	2016	Human	YuLin CDC	512	64	16	0	0.25	512	1	2	4	2	512	0.5	0.5	64	NA+CIP+OFX+AMP+SUL+TE
SH12SF064	2012	Food	ShangHai CDC	512	16	8	0.125	0.125	64	8	2	512	4	512	0.5	0.25	128	NA+CIP+OFX+AMP+C+SUL+TE
SH16G3333	2016	Human	YuLin CDC	512	32	16	0.125	1	512	1	2	256	256	512	0.5	1	128	NA+CIP+OFX+AMP+C+FFC+SUL+TE
GDYF1693	2016	Chicken	YunFu GuangDong	512	64	32	4	32	512	64	2	256	256	512	0.5	0.5	64	NA+CIP+OFX+CTX+AMP+CN+C+FFC+SUL+TE
YL14T352	2014	Human	YuLin CDC	512	16	8	0	0.125	0	8	1	512	4	512	0.5	0.25	128	NA+CIP+OFX+C+SUL+TE
SH16G993/#1	2016	Human	YuLin CDC	32	0.5	1	0.25	1	8	2	16	512	256	512	0.5	1	128	NA+C+FFC+SUL+TE

Table S2. Drug resistance genes and *Salmonella* multidrug-resistant genomic island 1 detected in strains of *Salmonella* Kentucky ST198

Strain	Gene about fluoroquinolones			SGI
	gyrA	*parC*	PMQR	
SH14G2115	Ser83Phe、Asp87Asn	Tyr62Ser、Ser85Ile	*aac(6′)-Ib-cr*	
SH16G2622		Tyr62Ser		
SH11SF316		Tyr62Ser	*aac(6′)-Ib-cr*	
SH14G1773	Ser83Phe	Tyr62Ser		
SH13G513	Ser83Phe	Tyr62Ser		
YL13T014	Ser83Phe	Tyr62Ser	*aac(6′)-Ib-cr*、*qnrS*	
2016THC5	Ser83Phe	Tyr62Ser	*aac(6′)-Ib-cr*	
SH10SF302	His78Asn、Ser83Phe	Tyr62Ser		
YL13T055	Ser83Phe	Tyr62Ser	*aac(6′)-Ib-cr*、*qnrS*	
GDYX1403	Ser83Phe	Tyr62Ser	*aac(6′)-Ib-cr*	
YL14T335	Ser83Phe	Tyr62Ser	*aac(6′)-Ib-cr*	
SH11G1256	Ser83Phe	Tyr62Ser	*aac(6′)-Ib-cr*、*qnrS*	
GDSG1524		Tyr62Ser		
GDYX0712	Ser83Phe	Tyr62Ser		SGI1-Ks
GDYX0714	Ser83Phe	Tyr62Ser		
SH15G1046	Ser83Phe、Asp87Asn	Tyr62Ser、Ser85Ile		SGI1-Ks
SH10G616	Ser83Phe	Tyr62Ser	*aac(6′)-Ib-cr*	
SH16G2899	Ser83Phe	Tyr62Ser		
SH16G3600/#1	Ser83Phe、Asp87Gly	Tyr62Ser、Ser85Ile		
YL13T110	Ser83Phe	Tyr62Ser	*aac(6′)-Ib-cr*、*qnrS*	
SH16G3319	Ser83Phe、Asp87Asn	Tyr62Ser、Ser85Ile	*aac(6′)-Ib-cr*	SGI1-Ks
SH16G3599	Ser83Phe、Asp87Gly	Tyr62Ser、Ser85Ile		SGI1-Ks
GDBY1128	Ser83Phe、Asp87Asn	Tyr62Ser、Ser85Ile	*oqxAB*、*aac(6′)-Ib-cr*、*qnrS*	
SH14G1899		Tyr62Ser		
YL14T387	Ser83Phe、Asp87Asn	Tyr62Ser、Ser85Ile		
SH16G1155	Ser83Phe、Asp87Asn	Tyr62Ser、Ser85Ile		SGI1-Ks
GDYX1508A	Ser83Phe	Tyr62Ser	*qnrB*	
GDBY1023	Ser83Phe、Asp87Asn	Tyr62Ser、Ser85Ile		SGI1-Ks
SH16G3561	Ser83Phe、Asp87Asn	Tyr62Ser、Ser85Ile		SGI1-Ks
SH16G922	Ser83Phe、Asp87Gly	Tyr62Ser、Ser85Ile		
SHG1008		Tyr62Ser、Ser85Ile		SGI1-Ks
SH16G3800		Tyr62Ser、Ser85Ile	*oqxAB*	
SH16G1490	Ser83Phe、Asp87Gly	Tyr62Ser、Ser85Ile		SGI1-Ps
GDTH1524	Ser83Phe、Asp87Asn、Asp87Gly	Tyr62Ser、Ser85Ile	*aac(6′)-Ib-cr*、*qnrB*	
SH16G762	Ser83Phe、Asp87Asn	Tyr62Ser		SGI1-Ks
YL14T296	Ser83Phe、Asp87Asn	Tyr62Ser、Ser85Ile		
GDTH1504	Ser83Phe、Asp87Asn	Tyr62Ser、Ser85Ile		
GDXY1502	Ser83Phe	Tyr62Ser		
SH13G1085	Ser83Phe、Asp87Asn	Tyr62Ser、Ser85Ile		
GDTH1603	Ser83Phe、Asp87Asn	Tyr62Ser、Ser85Ile		
SH12SF063	Ser83Phe、Asp87Asn	Tyr62Ser、Ser85Ile		SGI1-Ks
SH13G1441	Ser83Phe、Asp87Asn	Tyr62Ser、Ser85Ile		
YL13T063	Ser83Phe、Asp87Asn	Tyr62Ser、Ser85Ile		SGI1-Ks
YL13T071	Ser83Phe、Asp87Asn	Tyr62Ser、Ser85Ile		
YL13T276	Ser83Phe、Asp87Asn	Tyr62Ser、Ser85Ile		SGI1-Ks
SH16G2529	Ser83Phe、Asp87Tyr	Tyr62Ser、Ser85Ile		
SH12SF062	Ser83Phe、Asp87Asn	Tyr62Ser、Ser85Ile		
SH13G1438	Ser83Phe、Asp87Asn	Tyr62Ser、Ser85Ile		
GDSZ1607	Ser83Phe、Asp87Asn	Tyr62Ser、Ser85Ile	*aac(6′)-Ib-cr*	
GDSZ1608	Ser83Phe、Asp87Asn	Tyr62Ser、Ser85Ile	*aac(6′)-Ib-cr*	
GDSG1536	Ser83Phe	Tyr62Ser、Ser85Ile	*aac(6′)-Ib-cr*	SGI1-Ks
SH16G2599	Ser83Phe、Asp87Gly	Tyr62Ser、Ser85Ile		
SH16G3509	Ser83Phe、Asp87Asn	Tyr62Ser、Ser85Ile		SGI1-Ks
SH12SF228		Tyr62Ser		
SH16G2485	Ser83Phe、Asp87Asn	Tyr62Ser、Ser85Ile		
SH16G256	Ser83Phe、Asp87Gly	Tyr62Ser、Ser85Ile		SGI1-Ks
SH16G3589	Ser83Phe、Asp87Gly	Tyr62Ser、Ser85Ile		SGI1-Ks
SH16G3634	Ser83Phe、Asp87Asn	Tyr62Ser、Ser85Ile		
SH12SF064	His78Asn、Ser83Phe、Asp87Asn	Tyr62Ser、Ser85Ile	*aac(6′)-Ib-cr*	
SH16G3333	Ser83Phe、Asp87Asn	Tyr62Ser、Ser85Ile		SGI1-Ks
GDYF1693	Ser83Phe、Asp87Asn	Tyr62Ser、Ser85Ile	*oqxAB*、*aac(6′)-Ib-cr*	SGI1-Ks
YL14T352	Ser83Phe、Asp87Asn	Tyr62Ser、Ser85Ile	*aac(6′)-Ib-cr*、*qnrB*	
SH16G993/#1		Ser85Ile	*qnrB*	
